# The effect of alphacypermethrin-treated mesh protection against African horse sickness virus vectors on jet stall microclimate, clinical variables and faecal glucocorticoid metabolites of horses

**DOI:** 10.1186/s12917-017-1198-x

**Published:** 2017-09-09

**Authors:** Patrick Page, Andre Ganswindt, Johan Schoeman, Gert Venter, Alan Guthrie

**Affiliations:** 1 Department of Companion Animal Clinical Studies, Faculty of Veterinary Science, University of Pretoria, Private Bag X04, Onderstepoort, 0110 South Africa; 2 Endocrine Research Laboratory, Department of Anatomy and Physiology, Faculty of Veterinary Science, University of Pretoria, Private Bag X04, Onderstepoort, 0110 South Africa; 3 PVVD, ARC-Onderstepoort Veterinary Institute, Private Bag X05, Onderstepoort, 0110 South Africa; 4 Equine Research Centre, Faculty of Veterinary Science, University of Pretoria, Private Bag X04, Onderstepoort, 0110 South Africa

**Keywords:** Horse, Jet stall, Alphacypermethrin, Mesh, Climate, Faecal glucocorticoid metabolites

## Abstract

**Background:**

African horse sickness (AHS) is of importance to health and international trade in horses worldwide. During export from and transit through AHS endemic countries or zones, physical and chemical measures to protect horses from the vectors of AHS virus (AHSV) are recommended by the World Organization for Animal Health. Protection of containerized air transport systems for horses (jet stalls) with alphacypermethrin insecticide-treated high density polyethylene mesh is effective in reducing the *Culicoides* midge vector attack rate. In order to determine the effect of this mesh on jet stall ventilation and horse welfare under temperate climatic conditions, jet stall microclimate, clinical variables and faecal glucocorticoid metabolite (FGM) levels of 12 horses were monitored during overnight housing in either a treated or untreated stall in two blocks of a 2 × 3 randomized crossover design.

**Results:**

Temperature difference between the treated stall and outside was significantly higher than the difference between the untreated stall and outside at 1/15 time points only (*P* = 0.045, *r* = 0.70). Relative humidity (RH) difference between the treated stall and outside did not differ from the untreated stall and outside. Temperature and RH in the treated stall were highly and significantly correlated with outside temperature (*r* = 0.96, *P* < 0.001) and RH (*r* = 0.95, *P* < 0.001), respectively. No significant differences were detected between rectal temperatures, pulse and respiratory rates of horses in the treated stall compared to the untreated stall. Mean FGM concentrations for horses housed in the treated stall peaked earlier (24 h) and at a higher concentration than horses housed in the untreated stall (48 h), but were not significantly different from baseline. No significant difference was detected in FGM concentrations when the treated and untreated stall groups were compared at individual time points up to 72 h after exiting the jet stall.

**Conclusions:**

Alphacypermethrin-treated HDPE mesh could be used under temperate climatic conditions to protect horses in jet stalls against AHSV vectors, without compromising jet stall microclimate and horse welfare.

**Electronic supplementary material:**

The online version of this article (doi:10.1186/s12917-017-1198-x) contains supplementary material, which is available to authorized users.

## Background

African horse sickness (AHS) is an infectious, non-contagious, arthropod-borne viral disease that is of importance to health and international trade in equids [1, 2]. Intercontinental trade is a potential route whereby viruses such as AHS virus (AHSV) may be introduced into equid populations, either via movement of infected hosts or *Culicoides* midge vectors [2–6]. In order to reduce risk of AHSV transmission during export from or transit through AHS endemic countries or zones, measures of a physical and chemical nature have been recommended by the World Organisation for Animal Health to protect horses against AHSV vectors [7]. Alphacypermethrin insecticide-treated high density polyethylene (HDPE) mesh has a rapid insecticidal effect against *Culicoides* midges [8] and has recently been shown to significantly reduce the midge attack rate on horses housed in containerised air transport systems (jet stalls) [9].

While horses are frequently transported by air in jet stalls, there is limited published data on jet stall microclimate, stress indicators, or blood parameters related to the welfare of horses during air transport [10–14]. Stall environment and microclimate affects equine respiratory health in particular during air transport [15]. There are currently no published reports on the effects of physical or chemical vector protection measures, such as alphacypermethrin insecticide-treated HDPE mesh, applied to jet stalls on stall microclimate or stress indicators of horses.

Non-invasive means of assessing transport-associated stress in horses include monitoring of behavioural indices, heart rate and heart rate variability, salivary cortisol and faecal glucocorticoid metabolite (FGM) concentrations [11, 12, 14, 16–18]. Glucocorticoid metabolites in faeces increase approximately one day after an increase in blood cortisol concentrations in ponies [19, 20] and mainly reflect prolonged stress [16, 18, 21]. FGM concentrations have been reported for monitoring adrenocortical response to road transport [16–18] and sales consignment [22, 23], but not in response to jet stall housing or air transport of horses.

The aims of the study were to determine the effect of alphacypermethrin insecticide-treated HDPE mesh applied to commercial jet stalls on jet stall microclimate, clinical variables and FGM concentrations of horses housed under stationary jet stall, temperate climatic conditions.

## Methods

The study was approved by the Animal Ethics Committee of the University of Pretoria (Study V011–14).

## Animals

Twelve clinically healthy, adult horses (six geldings and six mares) of mixed breed (eight Thoroughbred, two Basuto, two Boerperd) with mean (range) age 11 (5–20) years, and body mass 482 (404–584) kg were included in the study. The horses were resident at the study site as part of the research herd. Other than being led into the stalls for subjective behaviour assessment one week prior to the start of the study, horses were not acclimatised to the stalls. Grass and alfalfa hay was fed ad libitum during data collection, and water was provided in buckets every 4 h. Wood shavings were used as bedding in the stalls. During overnight data collection herd mates were kept in adjacent paddocks in direct view of the front of the jet stalls to reduce group separation stress. The study horses were kept grouped with their herd mates in an adjacent grassed paddock during the day. Only routine activities were conducted with the horses from 1 week before until study completion.

## Study design

A randomised, crossover experimental study was performed from 28 to 30 April 2014 and from 5 to 7 May 2014 (late summer in the southern hemisphere). Horses were housed between 16 h00 and 06 h00 in two jet stalls, located 8.5 m apart in a grass paddock (58 m × 69 m), at the Faculty of Veterinary Science, Onderstepoort (25°38′51.42″S, 28°10′45.96″E, 1238 m above sea level).

The horses were grouped according to gender and ranked according to body weight, then assigned to same gender pairs to ensure comparable horse body weight housed in each stall each night. One of each gender pair of horses was randomly assigned to either a treated or untreated stall and monitored overnight in two experimental blocks of a 2 × 3 design. The first block consisted of three consecutive nights followed by a five day rest period. Treatments were then crossed over for the stalls and three consecutive nights of data collection for the second block conducted in the same sequence. Horses were therefore housed in the same gender pair and stall compartment for each block, but with the treatment crossed over.

### Jets stalls

Two commercial, non-collapsible, Federal Aviation Administration approved, 15.5 m^3^ (length 3.18 m, width 2.44 m, height 2.44 m) jet stalls^1^ were used. The inside of the stall had 3 adjacent compartments for housing horses, with a separate grooms’ compartment in the front for monitoring, feeding and providing water. During data collection horses were housed in the outer compartment of each stall with the middle compartment left unoccupied to facilitate access for clinical monitoring. When closed each stall had a 1.7 square meter rectangular opening above the front and rear ramps for ventilation.

### Jet stall treatment

One stall was treated with a black, 400 denier, knitted monofilament HDPE mesh^2^ with 0.3 mm hole size, impregnated with 20–40 mg/square meter alphacypermethrin^3^ for protection against *Culicoides* biting midges, as previously described [9]. The mesh was custom-made to fit over the stall in a tent-like fashion, with zip connectors for access located at the end panels. When fitted, the mesh covered the ventilation openings of the stall. No mesh was applied to the untreated stall.

## Data collection and analyses

### Clinical variables

The horses’ habitus, appetite and vital signs (rectal temperature,^4^ pulse and respiratory rates) were recorded before entering the jet stall (16 h00), and every 4 h while housed inside the stall.

### Climatic variables

Microclimate [temperature and relative humidity (RH)] inside each stall was monitored hourly with data loggers^5^ secured 1 m above the floor in the grooms’ compartment. Outside temperature and RH were monitored on a logger located 10 m away from the stalls. Data were downloaded with commercial software^6^ at the end of each treatment block. Additional outside climatic variables (wind speed, rainfall) were recorded hourly using a weather station and data logger^7^ located in an adjacent paddock.

### Faecal sample collection and analysis

Faecal samples were collected before entry into the jet stall (Baseline), 24, 48 and 72 h after exiting the jet stall. Faecal samples were collected by manual extraction from the rectum using a lubricated, plastic rectal glove. The faecal material was transferred into a 25 ml plastic specimen container, frozen within 4 h of collection, and kept at −20 °C until hormone extraction and assay.

Faecal samples were lyophilized, pulverized, and sifted [24]. Thereafter, 0.10–0.11 g of faecal powder was extracted using 3 ml of 80% ethanol in water. After vortexing for 15 min, the mixtures were centrifuged for 10 min at 1500 *g*, and the supernatants transferred into microcentrifuge tubes and stored at −20 °C until steroid analysis. FGM analysis was performed as previously described using a group-specific 11-oxoaetiocholanolone enzyme immunoassay, measuring 11,17-dioxoandrostanes [20, 25], shown to provide valid information on adrenocortical function in horses [21–23]. Serial dilutions of faecal extracts produced displacement curves parallel to the standard curve of the assay. The sensitivity of the assay determined at 90% binding was 2 ng/g dry faeces. Intra- and inter-assay coefficients of variation, determined by repeated measurement of high- and low-concentration quality controls, ranged between 1.9% - 6.6% and 6.2% - 10.2%, respectively. Cross-reactivity of the antibody used has been previously reported [25].

### Statistical analyses

Statistical analyses were performed with SPSS® Statistics.^8^ Data were assessed for normality by evaluating descriptive statistics, plotting histograms and performing the Kolmogorov-Smirnov test. All statistical tests were two-tailed at the 5% level of significance.

Differences between treated and untreated jet stall and outside climatic variables were computed and compared at hourly time points by Kruskal Wallis test. Where the test statistic was significant pairwise comparisons were conducted with *P*-values adjusted for multiple comparisons. Effect sizes were calculated for pairwise comparisons. Pearson’s correlation coefficient was calculated for the relationship between jet stall microclimate and outside climatic variables. Clinical variables were compared within each treatment group with Friedman’s ANOVA. Where the test statistic was significant, pairwise comparisons were conducted with *P*-values adjusted for multiple comparisons. Wilcoxon signed-rank tests were used to compare clinical variables between horses housed in the treated stall versus the untreated stall. Natural-log-transformed FGM concentrations were used for data analysis. FGM data within each treatment group were compared with one-way repeated measures ANOVA with post hoc Bonferroni adjustment of *P*-values for pairwise comparisons. The assumption of sphericity was confirmed with Mauchly’s test. Comparisons of FGM data between treatment groups were performed using paired samples t-tests.

## Results

### Climatic variables

Temperature and RH (median [interquartile range]) recorded in the treated stall, untreated stall and outside were 16.1 °C (12.1–20.3 °C), 14.1 °C (9.5–17.8 °C), 9.8 °C (5.6–14.7 °C) and 73.0% (63.4–77.4%), 77.8% (60.7–83.3%) and 86.6% (67.2–97.8%), respectively (Additional files 1 and 2). Mean (range) outside wind speed during the data collection period was 0.2 km/h (0–0.3 km/h) and no rain was recorded.

**Fig. 1:**
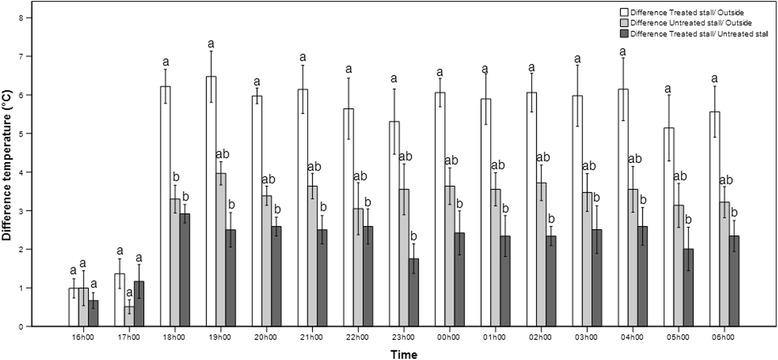
Hourly differences in temperature. Hourly differences (mean ± SEM) in temperature (°C) between a treated jet stall and outside, an untreated jet stall and outside, and a treated jet stall and an untreated jet stall recorded over 6 nights under temperate climatic conditions. Within each time point bars with a different lower case letter differ significantly (*P* < 0.05)

**Fig. 2:**
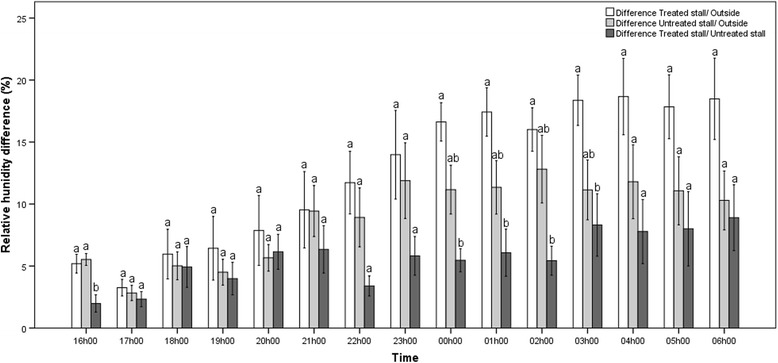
Hourly differences in relative humidity. Hourly differences (mean ± SEM) in relative humidity (%)between a treated jet stall and outside, an untreated jet stall and outside, and a treated jet stall and an untreated jet stall recorded over 6 nights under temperate climatic conditions. Within each time point bars with a different lower case letter differ significantly (*P* < 0.05)

Temperature difference between the treated stall and outside was significantly higher than the difference between the untreated stall and outside at the 18 h00 time point only (*P* = 0.045, *r* = 0.70), while temperature difference between the treated stall and outside was significantly higher than the difference between the treated stall and untreated stall at the majority of the time points (*P* = 0.001–0.041, *r* = 0.71–1.06) (Fig. 1). Relative humidity difference between the treated stall and outside did not differ from the difference between the untreated stall and outside at any time point, while RH difference between the treated stall and outside was significantly higher than the difference between the treated stall and untreated stall at few time points (*P* = 0.005–0.033, *r* = 0.73–0.91) (Fig. 2).

Temperature in the treated stall (*r* = 0.96, *n* = 90, *P* < 0.001) and untreated stall (*r* = 0.99, *n* = 90, *P* < 0.001) were highly and significantly correlated with outside temperature. RH in the treated stall (*r* = 0.95, *n* = 90, *P* < 0.001) and untreated stall (*r* = 0.97, *n* = 90, *P* < 0.001) were highly and significantly correlated with outside RH.

### Clinical variables

No adverse effects of housing in the treated or untreated stalls were recorded in any of the horses. All horses remained alert and responsive with subjectively normal appetite, water intake, regular faecal output and urination. Vital signs recorded in the treated and untreated groups before entering the stall and during overnight housing are summarised in Table 1 and Additional files 3, 4 and 5.

**Table 1:** Clinical variables (mean ± SD) of horses in the treated and untreated jet stall groups before entering the stall (16 h00) and during overnight housing (20 h00, 0 h00 and 04 h00)

	Group	Time point			
		16 h00	20 h00	0 h00	04 h00
Rectal temperature (°C)	Treated stall	37.8 ± 0.7	38.1 ± 0.7	37.8 ± 0.6	37.5 ± 0.7
	Untreated stall	37.7 ± 0.6	37.8 ± 0.6	37.6 ± 0.3	37.5 ± 0.3
Pulse (beats/min)	Treated stall	37 ± 9	40 ± 11	37 ± 7	37 ± 4
	Untreated stall	44 ± 11	38 ± 7	39 ± 9	35 ± 11
Respiration (breaths/min)	Treated stall	16 ± 7	21 ± 12	16 ± 5	14 ± 4
	Untreated stall	14 ± 3	15 ± 5	16 ± 6	14 ± 3

No significant differences were detected between the 4-hourly rectal temperatures, pulse and respiratory rates for the horses in the untreated stall. For the horses in the treated stall rectal temperature differed significantly for the 20 h00 compared to the 04 h00 time point only (Χ^2^_F_ = 11.283, df = 3, *P* = 0.01), while pulse and respiratory rates did not differ significantly between time points. There was no significant difference between rectal temperature, pulse and respiratory rates of horses in the treated stall compared to the untreated stall at individual time points.

### FGM concentrations

**Fig. 3:**
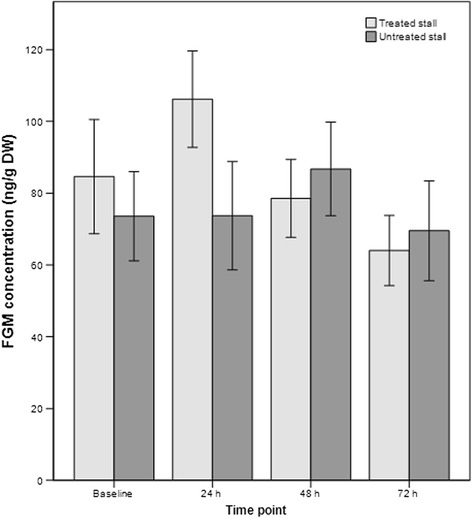
Faecal glucocorticoid metabolite concentrations. Faecal glucocorticoid metabolite concentrations (mean ± SEM) from horses housed in a treated and untreated jet stall, between 16 h00 and 06 h00 for 6 nights, before entrance into the stall (Baseline), 24, 48, and 72 h after exiting the stall.

An increase in mean FGM concentrations from baseline levels was detected in both treatment groups after overnight housing in the jet stalls (Fig. 3). Mean FGM concentrations for horses housed in the treated stall peaked earlier (24 h) and at a higher concentration than horses housed in the untreated stall (48 h). No significant difference was detected in FGM concentrations between baseline samples and samples obtained up to 72 h after exiting the stall for both the treated and untreated stall groups. No significant difference was detected in mean FGM concentrations when the treated and untreated stall groups were compared at individual time points.

## Discussion

Alphacypermethrin-treated HDPE mesh applied to commercial jet stalls as a physical and chemical protection barrier against AHSV vectors did not compromise jet stall microclimate, clinical variables or indicators of physiological stress in horses housed under stationary stall, temperate climatic conditions.

Closed top HMA-type jet stalls are commonly used to transport horses internationally. These stalls avoid potential fire alarm concerns associated with air conditioning units of climate-controlled stalls, however ventilation and microclimate could be affected by application of mesh around the stalls for protection against vectors. The outside temperatures in the present study were within or lower than a guideline range for transport of horses (10–21 °C), while the RH was higher than the recommended range (45–50%) [26], as expected for the overnight study conditions. Similarly, the outside and jet stall temperatures in the present study were within or lower than corresponding cargo hold and jet stall temperature ranges reported during air transit of horses, and the outside and jet stall RH was similar or higher than reported [10–14]. Marked differences in study conditions and the type of jet stall utilised limit direct comparison of microclimate between the present and the cited studies, however.

In order to better assess the impact of the mesh on microclimate, the differences computed between treated and untreated jet stall microclimate and outside climatic variables were analysed as outside conditions, which were highly correlated with those inside the jet stalls, varied daily making comparisons of differences more appropriate than actual values [27]. In the present study, while the temperature in the treated stall was consistently higher than the outside temperature, likely associated with retention of metabolic heat produced by the horses [28] and low overnight outside temperatures, the treated stall/ outside temperature difference was only significantly higher than untreated stall/ outside difference at one time point. The difference between the treated stall/ outside RH also did not differ significantly from the untreated stall/ outside RH. In fact, the treated stall RH was consistently lower than the outside RH despite anticipated evaporative heat loss which would be expected to increase RH [28]. These findings support no increased risk to thermoregulation with application of the HDPE mesh to a jet stall as compared to an untreated stall under similar climatic conditions.

While a thermal index limit of 28 °C is specified for equine competition there is currently no defined value to prevent equine thermal stress during equine transport [27] and further research has been recommended to improve thermal environment and ventilation of equine transport systems [29]. In the absence of data under more extreme environmental conditions for similar jet stalls protected with HDPE mesh, opening of the mesh flaps adjacent to stall ventilation points (e.g. above the front and back ramps) to promote ventilation is recommended as a precautionary measure if hot or humid environmental conditions arise during the transport process, such as during stopovers with exposure to ambient air [11]. Opening the mesh of stalls located at the rear of an aircraft first is recommended based on the temperature and RH gradient in the cargo hold whereby the stalls at the rear experience warmer, moister conditions, especially when the aircraft is stationary [10]. Furthermore, it is recommended that jet stall microclimate be monitored regularly during the transport process, particularly under more extreme environmental conditions when mesh protection is used. As emergency opening of the mesh to promote ventilation could increase risk of exposure to AHSV vectors, additional methods of protection such as insecticides or repellents applied to horses in the stalls or insecticidal aerosols sprayed in the cargo hold is advised.

Overnight housing in the treated jet stall had no significant effect on clinical variables compared to housing in an untreated stall. Horses were assessed under stationary jet stall field conditions in the present study which precluded confounding factors associated with air transport, such as loading into the aircraft, take off, turbulence, landing and unloading. Increased heart rates have been reported during these transitional events with return to resting values during level flight [11, 12, 14]. In the present study, respiratory rate also did not differ significantly between time points monitored in the treated stall, further supporting a lack of thermal stress as respiratory rate would be expected to increase [28] as a heat loss mechanism if the treated mesh compromised ventilation.

Though the effects of road transport [16–18] on cortisol release, heart rate and HRV have been reported for horses, there is limited information on the effect of air transport on stress indicators [11, 12, 14], and none have reported on the effect of jet stall transport on FGM levels. In contrast to monitoring of adrenocortical function by measurement of plasma and salivary cortisol, which reflect acute changes in cortisol release [30], FGM concentrations reflect only marked or prolonged increases in cortisol release with a lag time of about 24 h related to species-specific intestinal passage time in horses [17, 20, 21]. In the present study, which was limited to investigating the effects of stationary housing in a jet stall, no significant difference from baseline was detected in FGM concentrations, neither was there a significant difference between treatment groups. These findings, which are supported by the clinical variable data, indicate that stationary housing in an untreated jet stall or one protected with alphacypermethrin-treated HDPE mesh under temperate climate conditions is not associated with a significant stress response in horses. The earlier and higher peak in FGM concentrations for horses housed in the treated stall warrants further investigation in a larger cohort, however, but may be associated with other factors, e.g. mesh-associated impaired visual contact with herd mates compounding a herd group disruption effect [22].

Although caution is advised in practical application of the study findings based on the recognised study limitations including a stationary jet stall investigated under temperate field conditions, relatively low number of horses of varying breed, age, gender and temperament enrolled, and lack of a control group not housed in a jet stall, the findings support the safety of the HDPE mesh applied to comparable jet stalls under similar climatic conditions. Other meshes shown to protect housed horses against biting midges [31] could be investigated in a similar fashion.

## Conclusions

Alphacypermethrin-treated HDPE mesh applied to jet stalls housing horses had no adverse effect on jet stall microclimate, clinical variables or stress indicators of horses housed in the stalls under temperate climatic conditions. While this mesh could be used as a safe and effective physical and chemical method for protection of horses in jet stalls against *Culicoides* midges under similar temperate climatic conditions, further investigation is required under more extreme climatic conditions as well as during air transport.

## Additional files

Additional file 1: Temperature (°C; mean ± SEM) recorded at hourly time points in a treated jet stall, an untreated jet stall and outside over 6 nights under temperate climatic conditions. (BMP 1953 kb)

Additional file 2: Relative humidity (%; mean ± SEM) recorded at hourly time points in a treated jet stall, an untreated jet stall and outside over 6 nights under temperate climatic conditions. (BMP 1953 kb)

Additional file 3: Rectal temperature (°C; mean ± SEM) of horses in the treated and untreated jet stall groups before entering the stall (16 h00) and during overnight housing (20 h00, 0 h00 and 04 h00). (BMP 952 kb)

Additional file 4: Pulse rate (beats/ min; mean ± SEM) of horses in the treated and untreated jet stall groups before entering the stall (16 h00) and during overnight housing (20 h00, 0 h00 and 04 h00). (BMP 952 kb)

Additional file 5: Respiratory rate (breaths/ min; mean ± SEM) of horses in the treated and untreated jet stall groups before entering the stall (16 h00) and during overnight housing (20 h00, 0 h00 and 04 h00). (BMP 952 kb)

## Abbreviations

AHS: African horse sickness
AHSV: African horse sickness virus
ANOVA: Analysis of variance
FGM: Faecal glucocorticoid metabolite
HDPE: High density polyethylene
RH: Relative humidity

## Footnotes

1 KLM HMA jet stall, European Horse Services, Belgium

2 RK02 70% Shade Cloth, Alnet, South Africa

3 Fendona®6, BASF Agro BV Arnhem, Switzerland

4 Thermoval® rapid digital thermometer, Paul Hartmann AG, Heidenheim, Germany

5 iButton® DS1923, Fairbridge Technologies CC, Wendywood, South Africa

6 Coldchain Thermo Dynamics Software, Fairbridge Technologies CC, Wendywood, South Africa

7 Vantage Pro2 and Weatherlink, Davis, USA

8 SPSS® version 23, IBM, USA
