# Supplementary figures and images for: The effect of alphacypermethrin-treated mesh protection against African horse sickness virus vectors on jet stall microclimate, clinical variables and faecal glucocorticoid metabolites of horses
Source: BMC Vet Res. 2017 Sep 9;13:283. doi: 10.1186/s12917-017-1198-x (PMC5591536; doi:10.1186/s12917-017-1198-x)

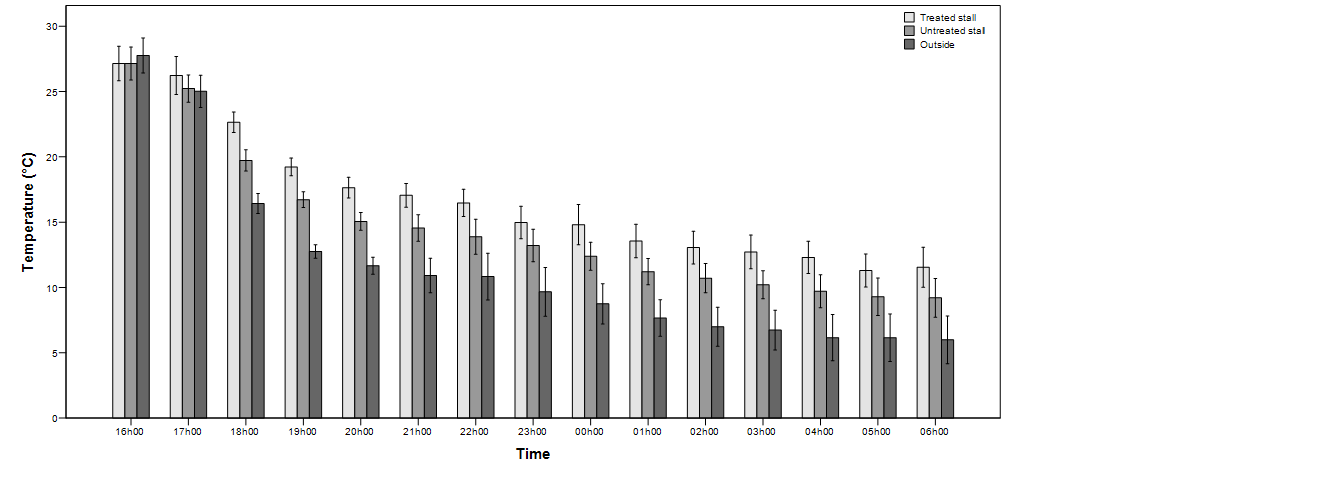

Supplement: Supplementary file 1 — Temperature (°C; mean ± SEM) recorded at hourly time points in a treated jet stall, an untreated jet stall and outside over 6 nights under temperate climatic conditions. (BMP 1953 kb) [file 12917_2017_1198_MOESM1_ESM.bmp]

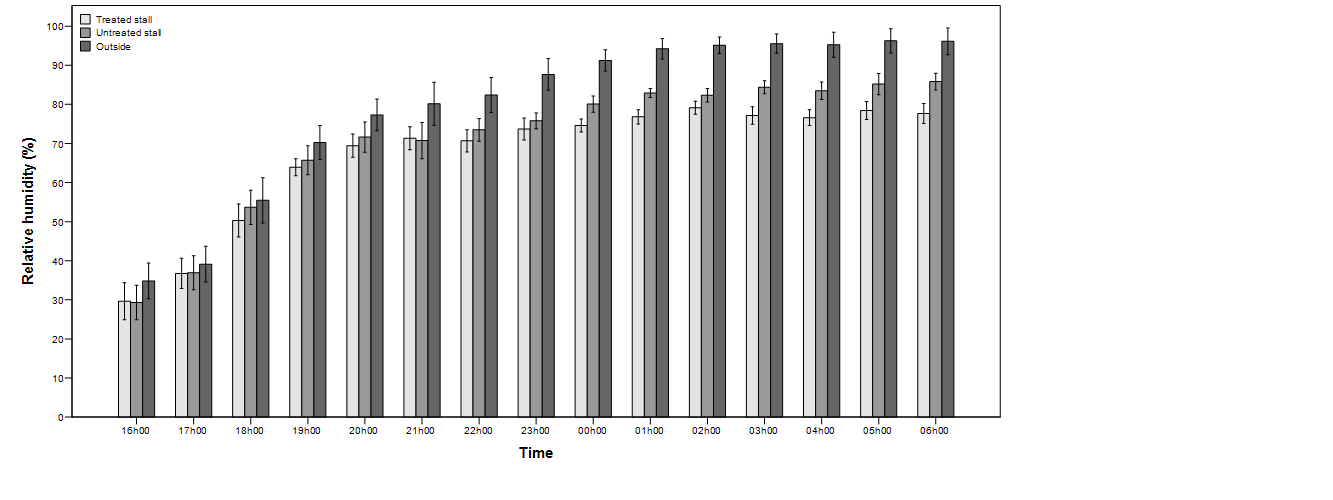

Supplement: Supplementary file 2 — Relative humidity (%; mean ± SEM) recorded at hourly time points in a treated jet stall, an untreated jet stall and outside over 6 nights under temperate climatic conditions. (BMP 1953 kb) [file 12917_2017_1198_MOESM2_ESM.bmp]

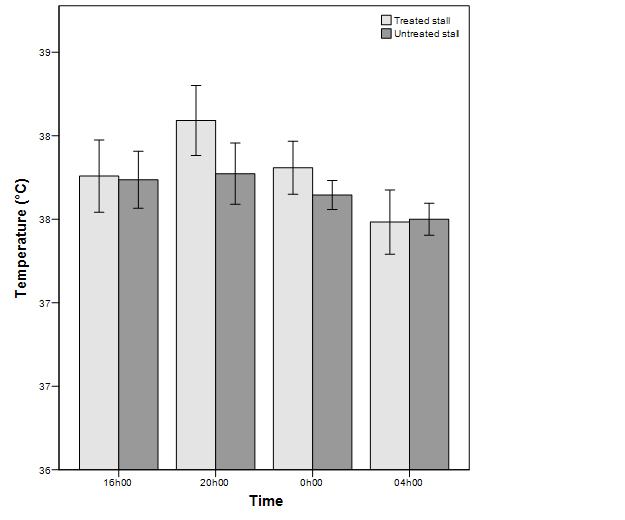

Supplement: Supplementary file 3 — Rectal temperature (°C; mean ± SEM) of horses in the treated and untreated jet stall groups before entering the stall (16 h00) and during overnight housing (20 h00, 0 h00 and 04 h00). (BMP 952 kb) [file 12917_2017_1198_MOESM3_ESM.bmp]

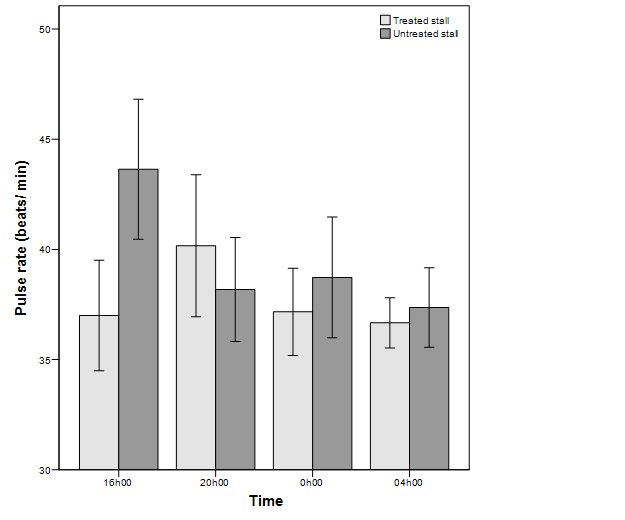

Supplement: Supplementary file 4 — Pulse rate (beats/ min; mean ± SEM) of horses in the treated and untreated jet stall groups before entering the stall (16 h00) and during overnight housing (20 h00, 0 h00 and 04 h00). (BMP 952 kb) [file 12917_2017_1198_MOESM4_ESM.bmp]

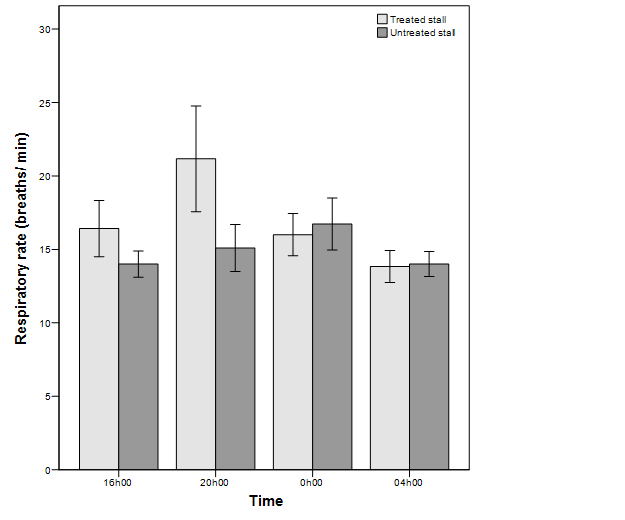

Supplement: Supplementary file 5 — Respiratory rate (breaths/ min; mean ± SEM) of horses in the treated and untreated jet stall groups before entering the stall (16 h00) and during overnight housing (20 h00, 0 h00 and 04 h00). (BMP 952 kb) [file 12917_2017_1198_MOESM5_ESM.bmp]
